# Editorial: SLC12A5-dependent neurological disorders

**DOI:** 10.3389/fnmol.2025.1611682

**Published:** 2025-05-08

**Authors:** Igor Medina, Amy McTague, Coralie Di Scala, Claudio Rivera

**Affiliations:** ^1^Aix-Marseille University, Mediterranean Institute of Neurobiology (INMED), French National Institute for Health and Medical Research (INSERM), Marseille, France; ^2^Zayed Centre for Research into Rare Disease in Children, Faculty of Population Health Sciences, University College London, London, United Kingdom; ^3^Neuroscience Center, HiLIFE, University of Helsinki, Helsinki, France

**Keywords:** SLC12A5, KCC2, NDD, epilepsy, chloride

The intricate choreography of neuronal communication relies on a delicate balance between excitation and inhibition. At the heart of this balance lies the potassium-chloride cotransporter KCC2, encoded by the SLC12A5 gene—a protein that has emerged as a molecular cornerstone of inhibitory neurotransmission in the central nervous system (CNS). The Research Topic “*SLC12A5-Dependent Neurological Disorders*” reflects a growing recognition that disruptions in chloride homeostasis, and in particular, KCC2 dysfunction, are central to a spectrum of devastating neurological disorders.

Since its discovery over two decades ago, KCC2 has been studied extensively for its role in shaping GABAergic and glycinergic inhibition (Payne et al., 1996; Rivera et al., 1999; Hübner et al., 2001). These early studies established its essential function in hyperpolarizing the chloride reversal potential, thereby enabling inhibitory neurotransmission. Yet, despite substantial advances, many questions remain unresolved, particularly regarding the diversity of its regulatory mechanisms and its broader roles in neurodevelopment and disease.

The articles in this Research Topic arise from the momentum generated by the first and second International Meetings on SLC12A5-Related Neurodevelopmental Disorders (SLC12A5-NDD), held in Marseille in 2023 and 2025. These meetings gathered experts from across the globe, united by a common goal: to untangle the biological complexity of KCC2 and accelerate translational efforts toward effective treatments. The scope of the discussions expanded from the specific role of KCC2 to the broader family of solute carrier (SLC) transporters, reflecting an evolving understanding of their collective influence on neuronal function.

Several key themes emerged across the contributions. First, compelling genotype-phenotype associations were described by Järvelä et al., illustrating how SLC12A5 mutations—particularly biallelic variants—can lead to severe epileptic encephalopathies such as epilepsy of infancy with migrating focal seizures (EIMFS). These findings highlight the potential for SLC12A5 variants to act not only as primary pathogenic drivers but also as genetic modifiers in a range of neurodevelopmental disorders, including autism spectrum disorder, Rett syndrome, Fragile X, and Dravet syndrome.

Second, efforts to dissect the molecular underpinnings of KCC2 regulation are yielding valuable insights. Uvarov et al. employed a transposon-based mutagenesis approach to identify novel C-terminal motifs in KCC2, uncovering mutants with altered chloride transport and surface expression. These discoveries pave the way for a more precise understanding of how structural domains govern KCC2 function, informing strategies for therapeutic modulation.

Kadam and Hegarty offered a comprehensive overview of the therapeutic landscape, advocating for the development of first-in-class KCC2 activators to address the unmet needs in epilepsy, neurodevelopmental disorders, and beyond.

In a significant extension of the field toward biomarker discovery and non-invasive diagnostic strategies, Caccialupi Da Prato et al. demonstrated that KCC2 expression in blood-derived exosomes can serve as a reliable peripheral marker of functional neuronal integrity. Using both neuronal and total exosome fractions isolated from blood serum in a mouse model of traumatic brain injury (TBI), they observed a persistent decrease in exosomal KCC2 levels, correlating with cognitive decline, depressive-like behaviors, altered network activity, and impaired secondary neurogenesis. These findings offer a compelling case for the use of exosomal KCC2 as both a prognostic and pharmacodynamic biomarker in post-traumatic and possibly neurodevelopmental or neuropsychiatric conditions.

Watanabe et al. explored the early establishment of GABAergic inhibition in hypothalamic CRH neurons. Their findings suggest that KCC2-mediated chloride extrusion occurs earlier in hypothalamic regions than in cortical areas, a result with potential implications for understanding stress regulation and vulnerability to early-life insults.

Together, these contributions underscore the multidimensional impact of KCC2 dysfunction and the urgent need for integrated research strategies. Moving forward, three priorities must guide the field: (i) implementing early diagnostic pathways for SLC-related disorders, (ii) deepening our mechanistic knowledge of chloride transport and regulation, and (iii) advancing therapeutic pipelines toward clinical translation—including pharmacological agents and reliable biomarkers such as exosomal KCC2.

As the field matures, a convergence of genetic, molecular, and translational neuroscience promises to reshape the clinical landscape for individuals affected by KCC2-related disorders. The community fostered through the SLC12A5-NDD meetings continues to grow, grounded in collaboration and shared purpose. With continued investment, both intellectual and infrastructural, the once-mysterious chloride transporter KCC2 may soon become the target of tomorrow's most effective neurological therapies.
